# Low-Load and High-Precision Forming Technology for Large-Scale Graded Doubly Curved Q890 High-Strength Steel Thick Plates

**DOI:** 10.3390/ma19132755

**Published:** 2026-06-29

**Authors:** Shuo Wang, Lin Zhu, Bingyan Jing, Yibo Su, Chunyu Ou, Yanli Lin, Yingguang Zhao, Changdi Ma, Zhubin He

**Affiliations:** 1 School of Mechanical Engineering, Dalian University of Technology, Dalian 116024, China; 2 State Key Laboratory of High-Performance Precision Manufacturing, Dalian 116024, China

**Keywords:** Q890 high-strength steel thick plates, graded double curvature, clamping force surge

## Abstract

Large-scale asymmetric doubly curved thick shells made of high-strength steel are key components in deep-sea and nuclear pressure vessels. Integral pressing is an important manufacturing method for such components, but it still faces two major challenges: excessive clamping force and poor springback predictability. To address these issues, this study conducts a combined experimental and numerical investigation on a 16 mm thick Q890 high-strength steel plate. First, the through-thickness plastic gradient and cyclic stress–strain response of the material were characterized, and a mixed hardening model incorporating through-thickness gradient plasticity was established. To suppress the lateral force induced by the asymmetric geometry, a blank positioning strategy was proposed, reducing the lateral force to below 2 t (Reduced by 65%). More critically, a striking phenomenon is revealed: during the final 1 mm of the clamping stroke, the forming force surges abruptly from approximately 680 t to 5090 t. Detailed analysis identifies the root cause as the synergistic effects of a sharp increase in contact area, a drastic rise in frictional resistance, and the onset of localized upsetting in regions already in contact with the die. To suppress the load surge while maintaining forming accuracy, a normal-direction over-compensation strategy was proposed. By deliberately increasing the normal compensation, the blank retains a bending-dominant deformation mode at the target clamping position, thereby avoiding the critical contact expansion, frictional buildup, and localized upsetting that trigger the force surge. Through iterative simulations, the optimal die surface is determined, achieving a forming force below 1000 t with a simulated shape deviation within 0.96 mm. Experimental validation using a purpose-built die on a 1000 t press successfully produces the shell with a maximum profile deviation of 1.67 mm, meeting high-accuracy requirements. This work establishes a new paradigm for low-load, high-accuracy forming of thick high-strength steel shells by actively managing contact evolution and deformation mode via normal-direction over-compensation, offering a practical pathway to one-shot tryout success for critical pressure hulls.

## 1. Introduction

Large-scale, asymmetrically variable doubly curved thick shells made of high-strength steel are critical load-bearing components in deep-sea submersibles, nuclear pressure vessels, and advanced defense equipment. The forming accuracy of these components directly affects manufacturing cost, service safety, and operational lifespan. Therefore, developing high-precision, high-efficiency forming technologies for such thick shells is of paramount engineering and scientific significance.

Traditional forming methods for high-strength steel curved shells include point-pressing, line-heating (water-bending), and laser bending. Despite their specific advantages, these methods suffer from inherent limitations: Multi-point forming is a discrete-die forming method in which a set of independently adjustable punches is used to approximate the target curved surface and form the plate into the desired shape. However, this method relies on a discrete punch array, which requires a complex and costly tooling system and is generally unsuitable for the bending forming of thick plates [1]. Line-heating forming is a thermal forming method in which a moving heat source and subsequent cooling are applied along predefined paths, generating local thermal expansion, cooling-induced shrinkage, and residual deformation in the plate to obtain the required curvature. Nevertheless, this method requires strict control of heating parameters and cooling rates, and it suffers from poor process stability and environmental pollution [2]; Laser bending is a die-less or low-die forming method in which a laser beam is used as a localized heat source to generate a non-uniform temperature field, thermal stress, and plastic deformation in metal plates. However, laser bending involves high equipment costs and is highly sensitive to sheet thickness and material properties, making it unsuitable for large thick plates [3]. More critically, all these methods exhibit strong dependence on operator experience, low productivity, and risk of material property degradation due to multiple or localized loading cycles. Consequently, they cannot meet the stringent requirements for consistency, high accuracy, and high safety of large thick-shell components.

To overcome these bottlenecks, integral pressing—a process that forms the entire complex curved surface in a single clamping stroke without moving the blank—has emerged as a promising alternative [4,5,6,7,8]. This method fundamentally avoids the accuracy loss and performance non-uniformity associated with discrete paths or local heating, offering controllable accuracy, high productivity, and good process stability. However, integral pressing faces two core challenges in practical application: low springback prediction accuracy that leads to costly iterative die tryouts, and excessively high clamping force that challenges press capacity and die life.

Accurate springback prediction has long been challenging in sheet metal forming. Springback results from the coupling of material properties, geometry, boundary conditions, and loading paths. With advances in elastoplastic theory and finite element methods, numerical simulation has become a primary tool for studying springback. The accuracy of constitutive models is crucial. For instance, Li et al. improved springback prediction for DP980 steel using the Y-U hardening model with Mises/Yld2000 yield criteria [9]. Ben Said et al. developed an anisotropic mixed hardening model, demonstrating that non-associated anisotropic models better capture plastic deformation [10]. Hou et al. compared various yield criteria and hardening models, finding that Barlat2000 combined with Yoshida–Uemori hardening significantly improves prediction [11]. Choi et al. evaluated four constitutive models for DP980 and TWIP980 steels in two-stage U-bending, showing that Y-U and HAH models yield higher accuracy [12]. However, most existing studies focus on thin sheets under simple bending (e.g., U- or V-bending). Constitutive model calibration and springback compensation for complex doubly curved thick shells remain underexplored.

Moreover, traditional studies often assume uniform through-thickness material properties. In reality, hot-rolled and cooled thick plates exhibit significant gradients in microstructure and mechanical properties along the thickness direction: the surface layers, due to rapid cooling, have higher strength but lower ductility, while the core shows opposite trends. This gradient significantly affects springback behavior in thick-plate bending, yet most existing studies neglect it, limiting prediction accuracy [13,14,15,16,17].

To address springback, researchers have attempted displacement compensation to modify die surfaces. Conventional compensation aims to make the blank fully contact the die at the end of the clamping stroke. However, such approaches do not consider the issue of clamping force magnitude, and in the case of thick high-strength steel shells, may lead to unacceptably high forming loads. Furthermore, the asymmetric geometry of such components introduces lateral forces during pressing, which can compromise die safety and are rarely addressed in existing compensation strategies.

Overall, although previous studies have provided valuable insights into constitutive modeling, springback prediction, and springback compensation, most of them have focused on thin sheets and have addressed these issues separately. In other words, existing studies have mainly investigated either springback prediction or springback compensation, while the coupled problems of springback control and excessively high forming load in thick-plate forming have not been fully resolved. For large-scale high-strength steel thick plates, the interactions among through-thickness gradient plasticity, asymmetric doubly curved geometry, lateral-force control, springback compensation, and forming-load reduction remain insufficiently understood. In particular, the sharp forming-load increase during the final die-closing stroke, as well as its relationship with contact evolution, frictional buildup, and localized upsetting, has not yet been clarified. This knowledge gap limits the direct application of conventional integral pressing to large-scale high-strength steel thick-shell components.

Therefore, a low-load, high-accuracy forming strategy for thick high-strength steel doubly curved shells is developed. Taking a 16 mm thick Q890 high-strength steel as the object of study, the through-thickness plastic gradients and Bauschinger effect are first characterized, and a Chaboche mixed hardening model that incorporates gradient plasticity is calibrated. A finite element model accounting for the through-thickness gradient of material properties is established. To mitigate the lateral force induced by the asymmetric geometry, a blank positioning strategy is proposed. The phenomenon of a sharp clamping force surge during the final stage of the stroke is then revealed and its mechanism is elucidated through contact and friction analysis. On this basis, a normal-direction compensation strategy is proposed to avoid the force surge while maintaining forming accuracy, and the optimal die surface is determined through iterative simulations. Finally, a dedicated die is designed and experimental validation is carried out. This work provides an experimentally validated, systematic solution for the precise die design of large thick high-strength steel shells, offering significant engineering value toward “one-shot tryout success”.

## 2. Materials, Heterogeneity and Constitutive Model

### 2.1. In-Plane Isotropy Validation

**Figure 1:**
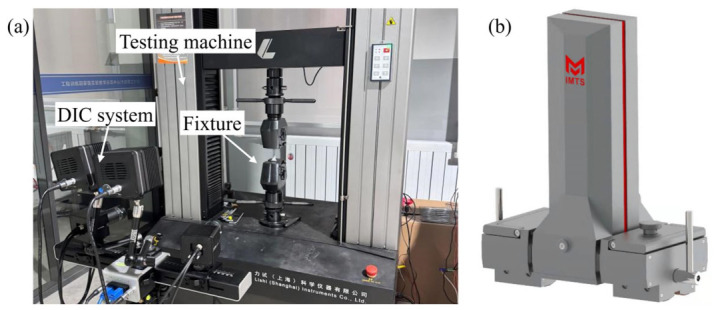
Mechanical testing equipment: (**a**) universal testing machine; (**b**) strength indentation tester.

The material used in this study was a 16 mm thick Q890 high-strength steel plate. To accurately predict the springback of large-scale high-strength steel doubly curved thick shells, it is essential to characterize the mechanical properties of the material with high accuracy. According to the Chinese standard GB/T 228.1–2010 [18], Metallic Materials—Tensile Testing—Part 1: Method of Test at Room Temperature, uniaxial tensile specimens were extracted from the same surface of the steel plate along the 0°, 45°, and 90° directions relative to the rolling direction. To ensure the reliability of the results, three specimens were prepared for each orientation. The uniaxial tensile tests were conducted using a LE5105 electronic universal testing machine (Lishi Scientific Instruments Co., Ltd., Shanghai, China). In addition, to further verify the accuracy of the results, indentation tests were performed using a strength indentation tester (Chengdu Weilitesi Technology Co., Ltd., Chengdu, China) at different locations on the plate surface to characterize the in-plane strength distribution. The mechanical testing equipment is shown in Figure 1. Figure 1a shows the digital image correlation (DIC) system, which was used for strain analysis during the mechanical property tests. Figure 1b shows the strength indentation tester, which was used for the nondestructive characterization of the in-plane strength distribution of the steel plate. These experiments provided the elastic parameters, initial yield stress, and the complete isotropic hardening curve of the material, which served as the fundamental data for the calibration of the isotropic hardening parameters.

**Figure 2:**
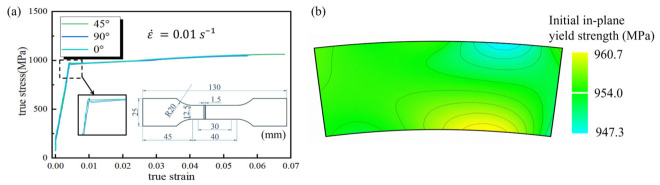
Mechanical test results of Q890 high-strength steel: (**a**) true stress–strain curves of Q890 high-strength steel; (**b**) yield strength contour map of Q890 high-strength steel.

Figure 2 shows the mechanical test results of the Q890 high-strength steel. Figure 2a presents the true stress–strain curves in different in-plane directions, which were obtained using a universal testing machine. Figure 2b shows the initial in-plane yield strength distribution of the Q890 high-strength steel blank, which was obtained using a strength indentation tester. It can be observed that the overall trends of the stress–strain curves are highly consistent. No significant differences are observed in either the yielding stage or the subsequent strain-hardening stage. The curves remain close to each other near the yield point and during the uniform deformation stage, with only minor differences in peak stress and strain-hardening rate. In addition, the strength variation among different measurement locations on the plate surface is relatively small, with a maximum strength of 960.7 MPa and a minimum strength of 947.3 MPa. The maximum fluctuation is only 13.4 MPa, accounting for approximately 1.4% of the average strength. These results indicate that the mechanical properties of the material are relatively uniform in the in-plane direction, with only slight fluctuations. This is mainly attributed to the large plate dimensions, which inevitably introduce a certain degree of in-plane property variation. Nevertheless, the material overall exhibits approximately isotropic yielding behavior. The initial yield strength, ultimate tensile strength, and elongation of the material are 969 MPa, 1003 MPa, and 7.12%, respectively. Moreover, the yield ratio is close to 1, indicating that the material possesses a relatively strong springback tendency.

### 2.2. Through-Thickness Gradient Characterization

**Figure 3:**
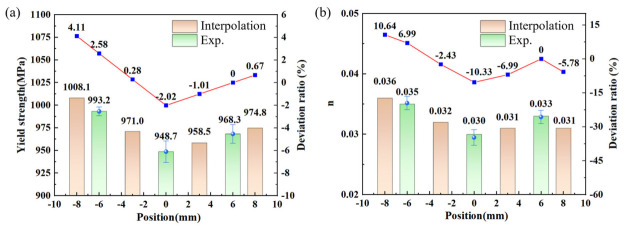
Through-thickness comparison of mechanical properties: (**a**) yield strength distribution along the thickness direction; (**b**) strain hardening exponent (n-value) distribution along the thickness direction.

Although the material exhibits relatively good uniformity in the in-plane direction, for a 16 mm thick high-strength steel plate, the hot-rolling and subsequent cooling processes inevitably lead to microstructural inhomogeneity along the thickness direction, resulting in a gradient distribution of mechanical properties. To accurately characterize this gradient, the plate was divided into three regions along the thickness direction, namely the surface layer, middle layer, and bottom layer, from which specimens were extracted separately. The specimen geometry was consistent with that used in the previous section. The mechanical properties of each layer, including yield strength and hardening exponent, were obtained through uniaxial tensile tests. To ensure continuity in the transition of properties between adjacent layers, interpolation processing was performed on the experimental data. The final results are presented in Figure 3 and Table 1.

As shown in Figure 3 and Table 1, the mechanical properties of the Q890 high-strength steel thick plate exhibit significant variation along the thickness direction. The yield strength shows a non-monotonic variation from approximately 948.74 MPa to 1008.07 MPa, indicating that the material properties are not uniformly distributed through the thickness. This difference leads to an asymmetric stress distribution along the thickness direction during forming, which causes a shift in the neutral layer position and further affects the springback behavior and its spatial distribution. Therefore, introducing the through-thickness gradient of material properties into springback prediction and compensation analysis is essential for more realistically describing the actual deformation characteristics and improving the accuracy and engineering reliability of the simulation results.

### 2.3. Cyclic Tension-Compression Test and Chaboche Mixed Hardening Model

Previous studies have shown that Q890 steel exhibits a pronounced Bauschinger effect, which is attributed to the accumulation of back stresses induced by dispersed carbides in its tempered sorbitic microstructure [19,20,21,22,23,24]. During the forming of graded doubly curved thick plates, the outer layers of the plate mainly experience tensile stress during loading, while they are subjected to compressive stress during unloading, corresponding to a reverse loading path. Under such cyclic loading conditions, the material exhibits a significant Bauschinger effect, which has a critical influence on the accuracy of springback prediction. Therefore, to accurately describe the reverse loading behavior of the sheet during bending deformation, cyclic loading tests were conducted to calibrate the kinematic hardening parameters in the mixed hardening model, thereby improving the accuracy of springback prediction.

**Figure 4:**
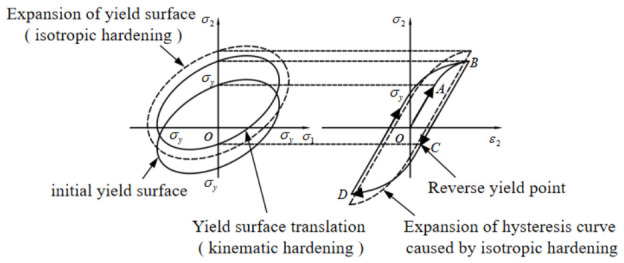
Schematic diagram of the mixed hardening model.

To further capture the Bauschinger effect and transient hardening behavior during reverse loading, the Chaboche mixed hardening model is used in this study. In this model, the total hardening is decomposed into the superposition of isotropic hardening and kinematic hardening components, as schematically illustrated in Figure 4.

The constitutive formulation of the model can be expressed as:

(1) $$f=\sqrt{\left[\frac{3}{2}\left(\sigma^{\prime }-\alpha^{\prime }\right):\left(\sigma^{\prime }-\alpha^{\prime }\right)\right]}-\sigma_{y0}-r\left(p\right)$$

where $\sigma^{\prime }$ is the deviatoric stress tensor, $\alpha^{\prime }$ is the back stress associated with kinematic hardening, $\sigma_{y0}$ is the initial yield stress, and $r\left(p\right)$ is the isotropic hardening function.

For nonlinear isotropic hardening, the function $r\left(p\right)$ is expressed as:

(2) $$r\left(p\right)=Q\left(1-e^{-bp}\right)$$

where Q and b are material parameters, Q can be determined from the difference between the peak stress and the initial yield stress, b controls the rate at which the stress approaches the saturation value, p and is the accumulated plastic strain.

For the kinematic hardening component, the increment of back stress dα in nonlinear kinematic hardening can be expressed as:

(3) $$d\alpha =\frac{2}{3}cd\varepsilon^{p}-\gamma \alpha dp$$

where c and γ are material parameters, $d\varepsilon^{p}$ is the plastic strain increment, and dp is the equivalent plastic strain increment. Under uniaxial loading conditions, the two are equivalent.

To improve the model accuracy, the Chaboche mixed hardening model can be represented by the superposition of multiple back stress components, as expressed by:

(4) $$\alpha =\sum_{k=1}^{N}\alpha_{k}$$

where N is the number of back stress components.

**Figure 5:**
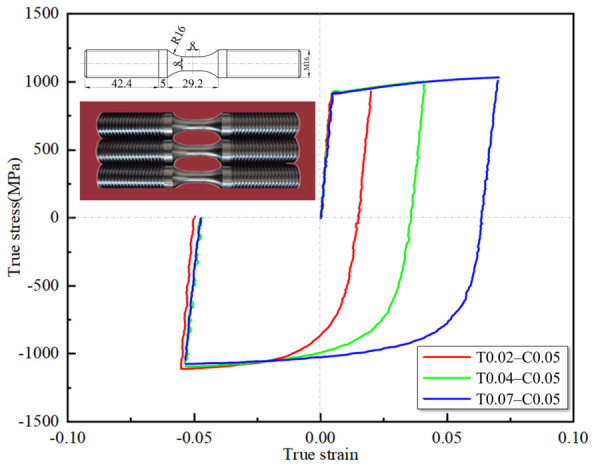
Cyclic tension–compression stress–strain curves.

The cyclic loading tests were conducted using cylindrical bar specimens on the experimental setup shown in Figure 1a. The gauge section of the specimens had a diameter of d = 8 mm and a gauge length of L = 8 mm. Strain-controlled symmetric tension–compression cyclic loading was applied. To better characterize the actual strain levels experienced by the material during the forming of doubly curved thick plates, cyclic tension–compression tests were conducted under different tensile pre-strain levels. Three test conditions were performed: (i) tensile strain of 0.02 followed by reverse compressive strain of 0.05 (T0.02–C0.05); (ii) tensile strain of 0.04 followed by reverse compressive strain of 0.05 (T0.04–C0.05); and (iii) tensile strain of 0.07 followed by reverse compressive strain of 0.05 (T0.07–C0.05). The obtained cyclic stress–strain curves are presented in Figure 5.

To separate the isotropic hardening component, the contribution of back stress needs to be subtracted from the total stress. The resulting isotropic hardening stress is denoted as ${\sigma_{i}}^{0}$, and the corresponding accumulated plastic strain is denoted as ${\varepsilon_{i}}^{pl}$. The cyclic loading tests include three loading cycles, yielding three sets of (${\sigma_{i}}^{0}$,${\varepsilon_{i}}^{pl}$) data. The parameter r(p) in Equation (2) corresponds to the difference between ${\sigma_{i}}^{0}$ and the initial yield stress, where p equals ${\varepsilon_{i}}^{pl}$. Since the initial yield stress must be included, the corresponding data for r(p) and p can be obtained from Figure 5. Based on the experimental data, Q is determined as −190 MPa, and the parameter b is obtained by least squares fitting. Thus, the isotropic hardening function is expressed as:

(5) $$r(p)=-190\left(1-e^{-0.85p}\right)$$

For the determination of the kinematic hardening parameters c and γ, the equivalent stress–strain data and back stress of Q890 high-strength steel are input into Abaqus 6.14, from which the parameters can be identified. The final Chaboche mixed hardening model parameters for Q890 high-strength steel are listed in Table 2.

**Figure 6:**
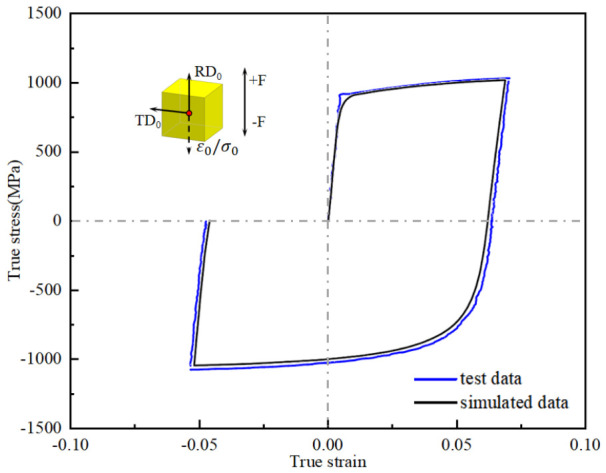
Comparison between the mixed hardening model predictions and experimental results.

To verify the capability of the calibrated Chaboche mixed hardening model in describing the cyclic plastic behavior of Q890 high-strength steel, a single-element cyclic loading finite element model was established in Abaqus 6.14. The same strain-controlled loading path as used in the experiments was applied in the simulation. The simulated stress–strain response was compared with the experimental results, as shown in Figure 6. It can be observed that the model accurately captures the yielding behavior during the initial tensile loading stage, the reduction in yield stress induced by the Bauschinger effect during reverse loading, and the subsequent hardening saturation trend in cyclic deformation. Quantitative comparison indicates that the prediction error of the peak stress is less than 5%, while the deviation in reverse yield stress is controlled within 3%. These results confirm that the calibrated model can effectively describe the cyclic plastic behavior of Q890 high-strength steel.

## 3. Finite Element Modeling and Discovery of Force Surge at Final Stroke

### 3.1. Finite Element Modeling

This section establishes a finite element model for the integral forming of a large-scale graded doubly curved thick shell component based on the calibrated Chaboche mixed hardening model and the through-thickness material property data described above, and systematically conducts springback prediction simulations.

**Figure 7:**
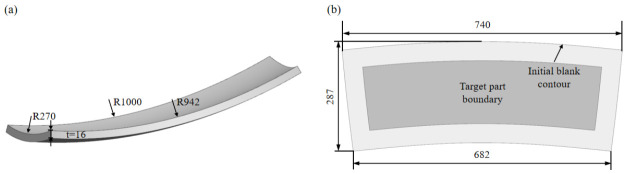
Geometry and dimensions of the target component and initial blank (unit: mm): (**a**) target component geometry and dimensions; (**b**) initial blank shape.

The target component is a typical non-developable doubly curved surface. Its geometric characteristics and the corresponding flattened initial blank shape are shown in Figure 7. The circumferential seam at the large end has an arc length of approximately 727 mm with a chord height of 66 mm, while the circumferential seam at the small end has an arc length of approximately 684 mm with a chord height of 60 mm. The longitudinal seam has an arc length of approximately 268 mm with a chord height of 31 mm. The plate thickness is 16 mm. Based on the neutral-layer geometric unfolding principle, the initial blank geometry is obtained. The chord length at the large end is 740 mm, the chord length at the small end is 682 mm, and the longitudinal height is 287 mm.

**Figure 8:**
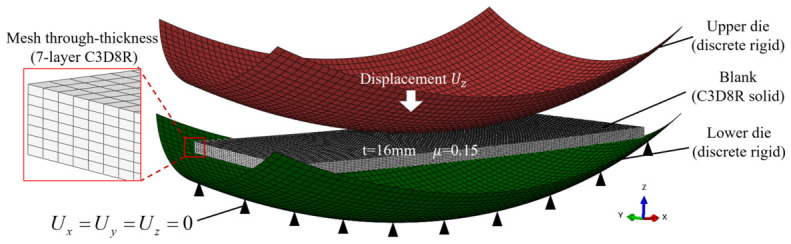
Finite element simulation model.

The finite element model was developed using Abaqus 6.14. The overall finite element model is shown in Figure 8. The upper and lower dies were defined as discrete rigid bodies and meshed using R3D4 elements, with an element size of 24 mm. The blank was defined as a three-dimensional deformable solid part and discretized using C3D8R elements, and the total number of elements in the blank was 37,170. The in-plane mesh size was set to 12 mm, and seven element layers were defined through the thickness to represent the material property gradient. In order to improve the computational accuracy in regions with large deformation, local mesh refinement was applied in high-strain areas. A surface-to-surface contact algorithm was adopted to describe the interaction between the tools and the blank, and the friction coefficient was set to 0.15. The lower die was fully fixed. For the upper die, all degrees of freedom except the translational displacement in the Z direction were constrained. The forming process was performed by imposing a prescribed displacement along the Z direction on the upper die. The displacement boundary condition was applied using a smooth amplitude curve to avoid sudden loading. The blank was not directly constrained and was allowed to deform freely under the contact interaction with the dies. Subsequently, an unloading step was performed to obtain the final geometry after springback.

The material parameters were defined based on the calibrated Chaboche mixed hardening parameters and the through-thickness layered mechanical property data. The plate thickness was discretized into seven layers, and the stress–strain data of each layer, obtained from uniaxial tensile tests and interpolation as described above, were assigned to the corresponding layers in the finite element model. For all layers, the Young’s modulus was set to 203 GPa, the Poisson’s ratio was 0.3, and the material density was 7.8 g/cm^3^. An identical mixed hardening constitutive framework was adopted for each layer. The hardening parameters were determined based on the results of cyclic tension–compression tests. In addition, the yield strength and strain hardening exponent of each layer obtained from uniaxial tensile tests were used to perform a layer-wise modification. The kinematic hardening parameters c and γ were kept constant for all layers, while only the initial yield stress and the n-value were adjusted to reflect the through-thickness variation.

### 3.2. Methods to Reduce Lateral Force During the Forming Process

**Figure 9:**
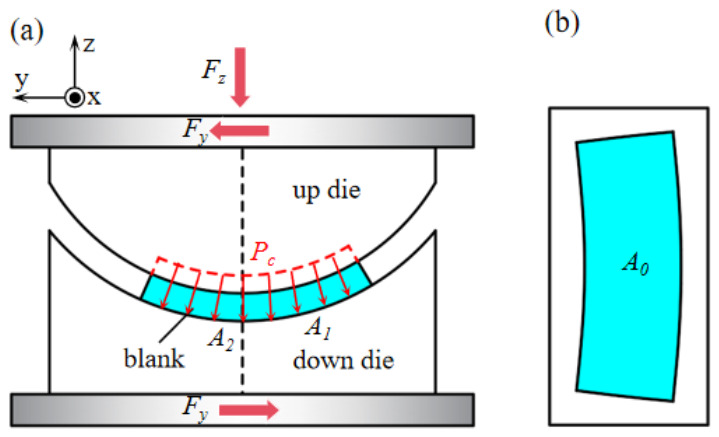
Schematic diagram of die lateral force: (**a**) overall lateral force acting on the die; (**b**) schematic illustration of blank positioning within the die.

Due to the pronounced asymmetric geometry of the blank in the longitudinal direction (large-end/small-end configuration), improper positioning within the die can easily induce significant lateral forces during the forming process, which may adversely affect the safe operation of the tooling system. Figure 9 illustrates the schematic distribution of the overall lateral force acting on the die. Along the die centerline, the blank can be divided into two regions, denoted as A1 and A2.

(6) $$P_{c}=F_{z}/A_{0}$$

where $F_{z}$ denotes the clamping force applied by the upper die, and $P_{c}$ represents the average contact load.

(7) $$F_{y1}=A_{1}\times P_{c}$$

(8) $$F_{y2}=A_{2}\times P_{c}$$

where $F_{y1}$ and $F_{y_{2}}$ denote the reaction forces exerted by regions A1 and A2 of the blank on the die.

(9) $$F_{y}=\left|F_{y_{1}}-F_{y_{2}}\right|$$

where $F_{y}$ denotes the overall lateral force acting on the die.

It can be observed that, in order to reduce the lateral force, the projected areas of regions A1 and A2 should be made as equal as possible.

**Figure 10:**
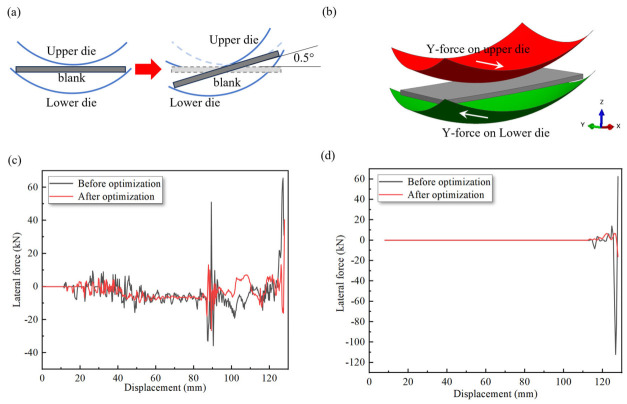
Model positioning and variation in lateral force: (**a**) schematic illustration of model position adjustment; (**b**) schematic of lateral force direction; (**c**) comparison of lateral force during die closing before and after optimization; (**d**) comparison of lateral force during die opening before and after optimization.

The analysis of the lateral force indicates that, due to the pronounced geometric asymmetry of the blank in the longitudinal direction (large-end/small-end configuration), improper positioning within the die can easily generate significant lateral forces, thereby adversely affecting the safe operation of the forming equipment. To achieve a more balanced distribution of projected areas between regions A1 and A2, a global rotation of the blank by 0.5° was introduced to reduce the lateral force. Figure 10 illustrates the blank position before and after optimization, as well as the corresponding evolution of the lateral force, where a positive value of lateral force indicates a direction toward the small-end side. It can be observed that, during the initial stage of upper die closure, the lateral force exhibits certain fluctuations in direction. As the die approaches full closure, the lateral force gradually shifts toward the small-end side, reaching a maximum value of approximately 6.3 t. During the unloading (die opening) stage, the force direction reverses toward the large-end side, with a peak value of approximately 11.7 t, and then gradually decreases to zero as opening continues. After optimization, the maximum lateral force during the closing stage is reduced to 4 t, while the peak lateral force during the opening stage is reduced to 1.6 t. This significant reduction effectively improves the stability of the forming process and greatly decreases the risk of die damage during experimental operation.

### 3.3. Analysis of the Final-Stroke Forming Load Surg

**Figure 11:**
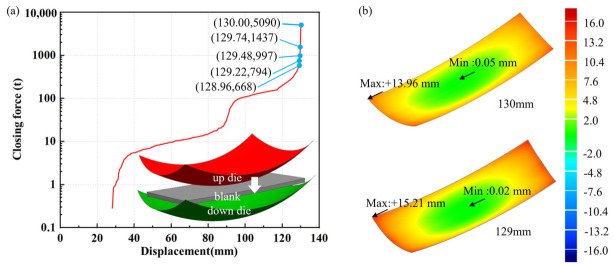
Clamping force curve and deviation comparison: (**a**) clamping force–displacement curve; (**b**) comparison of form deviation between 130 mm (fully closed) and 129 mm clamping displacement conditions.

Figure 11 presents the clamping force–displacement curve and the corresponding deviation comparison of the formed components under different displacement conditions during the pressing process of the large-scale graded doubly curved thick plate. It can be observed that a sharp increase in forming force occurs within the final 1 mm stroke of the full closure stage (at 130 mm clamping displacement), where the pressing force abruptly rises from approximately 680 t to 5090 t. By comparing the formed parts obtained at 130 mm (fully closed) and 129 mm clamping conditions with the target geometry, it can be seen that, although the clamping force differs by more than 4000 t, the difference in forming accuracy is relatively small. Specifically, the proportion of deviation within the range of −2 to 2 mm differs by only 1.27%.

To elucidate the mechanism responsible for the sharp increase in clamping force during the fully closed stage, the contact area, frictional shear stress, and thickness distribution of the blank were extracted at clamping displacements of 120 mm, 125 mm, 129 mm, and 130 mm (fully closed), respectively.

**Figure 12:**
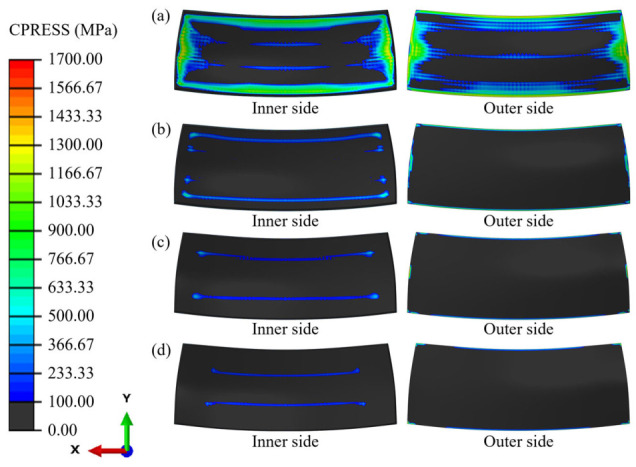
Contact area distributions on the inner and outer surfaces of the blank under different die-closing displacements: (**a**) 130 mm; (**b**) 129 mm; (**c**) 125 mm; (**d**) 120 mm.

Figure 12 compares the contact regions between the blank and the die under different clamping displacements. In the 120–129 mm stage, contact between the sheet and the die is mainly concentrated in the edge regions of the blank, with a relatively limited contact area. The blank still retains sufficient free deformation space and can accommodate deformation through local bending; therefore, the overall normal contact force remains relatively small, and the clamping force increases gradually. As the clamping displacement increases to 130 mm, full conformity between the blank and the die is achieved. The contact state transforms from partial contact to full-area surface contact, resulting in a significant increase in normal contact force, which in turn leads to a pronounced surge in clamping force.

**Figure 13:**
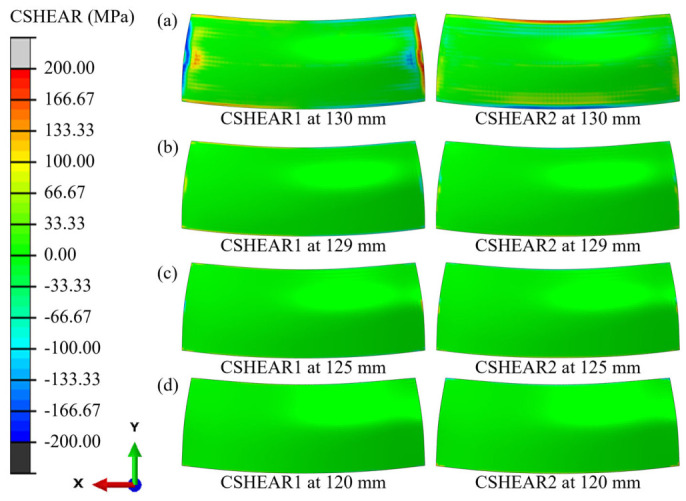
Frictional shear stress distributions between the blank and the die under different clamping displacements: (**a**) 130 mm; (**b**) 129 mm; (**c**) 125 mm; (**d**) 120 mm.

Figure 13 compares the frictional shear stress between the blank and the die under different clamping displacements. In the 120–129 mm stage, the sheet can undergo relative sliding along the die surface, and both the frictional shear stress and frictional resistance remain at relatively low levels. As the clamping displacement increases to 130 mm, the free sliding capability of the blank is significantly restricted. In local regions, the contact state gradually transitions from sliding friction to an approximately sticking (adhesive) condition, leading to a peak in frictional shear stress. To overcome this substantially increased resistance, a higher clamping load is required, which further intensifies the surge in clamping force.

**Figure 14:**
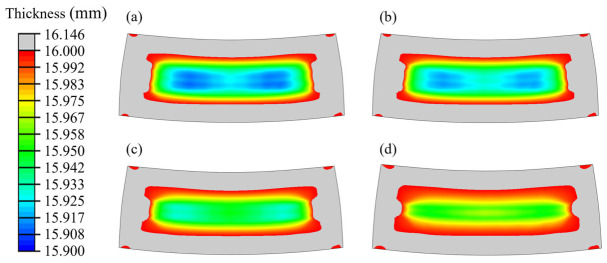
Thickness distributions of the blank under different clamping displacements: (**a**) 130 mm; (**b**) 129 mm; (**c**) 125 mm; (**d**) 120 mm.

Figure 14 shows the wall thickness distributions of the blank under different clamping displacements. In the 120–129 mm stage, the deformation of the blank is dominated by bending, accompanied by the initial onset of localized upsetting. As the clamping displacement increases to 130 mm, the highly constrained central region of the material is subjected to intense localized upsetting, resulting in pronounced thinning. According to the principle of volume constancy, the material in this upset region is extruded laterally and accumulates toward the less constrained outer regions. This upsetting-induced peripheral expansion significantly increases the contact area between the blank and the die and intensifies the geometric constraint. Consequently, both frictional shear stress and reaction forces increase rapidly, ultimately leading to a sharp surge in the clamping force.

In summary, the sharp increase in clamping force at the fully closed stage (130 mm) is the result of the combined effects of a significant expansion of the contact area, a rapid increase in frictional shear stress, and localized upsetting deformation. An excessively high clamping force not only poses a potential threat to the service life of the equipment and die, but may also lead to excessive local indentation or stress concentration within the formed component. Therefore, it is necessary to ensure the forming accuracy while simultaneously avoiding excessive clamping forces that could cause damage to the die system.

## 4. Low-Load Strategy: Normal-Displacement Compensation for “Bending Instead of Stretching”

As shown in Section 3.3, due to the changes in deformation mode, die–blank contact state, and frictional conditions during the final stage of the pressing process, the clamping force increases sharply when the clamping gap is 1 mm and under the fully closed condition. Although the required forming force increases by approximately 6.5 times (from 680 t to 5090 t), the maximum deviation of the formed component after springback is only 1.25 mm. This indicates that a significant increase in load does not necessarily correspond to a substantial improvement in forming accuracy. Based on this observation, a forming strategy based on adjusting the normal-direction compensation is proposed to achieve low-load forming. The core idea of the proposed method is to build upon the conventional springback compensation approach by appropriately increasing the normal-direction compensation. In this way, the sheet is formed into the target geometry while maintaining a certain clamping gap, ensuring that the deformation of the sheet remains dominated by bending. Consequently, the material is prevented from entering the high-force surge regime, thereby achieving a reduction in forming load.

Under the same 1 mm clamping gap condition, the die surface obtained after springback compensation exhibits a reduced local curvature radius, which significantly increases the contact pressure and deformation resistance between the blank and the die, resulting in a noticeably higher clamping force compared with the unmodified compensated die. To ensure forming accuracy while reducing the load on the equipment, the springback response obtained under the 1 mm clamping gap condition is first used as the basis for displacement compensation. On this basis, the normal-direction modification magnitude is further increased. Through this strategy, the sheet can achieve the required geometric accuracy under a 2 mm clamping gap condition, thereby effectively avoiding the clamping force surge associated with full die closure and realizing a coordinated optimization of forming accuracy and load level.

**Figure 15:**
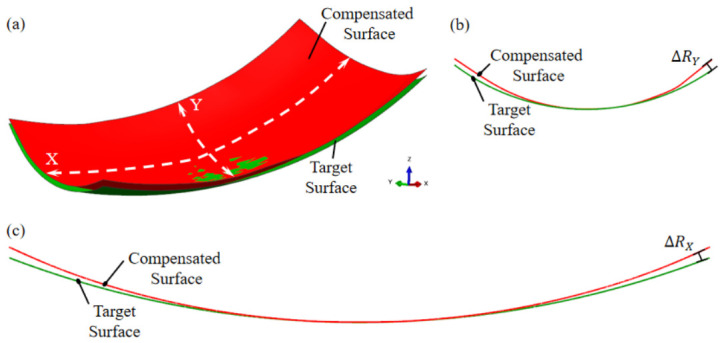
Comparison between the compensated surface and the target surface: (**a**) 3D comparison of the compensated die surface obtained after multiple iterations and the target surface; (**b**) comparison of Y-direction sectional profiles between the compensated surface and the target surface after multiple iterations; (**c**) comparison of X-direction sectional profiles between the compensated surface and the target surface after multiple iterations.

Due to the asymmetric geometry of the blank, the springback deviation is non-uniformly distributed: regions near the center of the geometry exhibit smaller springback, while edge regions exhibit larger springback. Therefore, a spatially varying displacement compensation method is adopted according to the position of the blank. The displacement compensation method corrects the die surface by moving nodes along the surface normal direction, and its mathematical expression is given as follows:

(10) $$M^{k+1}=M^{k}+\omega \cdot \left(T-P^{k}\right)$$

where $M^{k}$ and $M^{k+1}$ are the nodal coordinate vectors of the die surface at the k-th and (k + 1)-th iterations, respectively, and $T-P^{k}$ is the deviation vector at the k-th iteration. Through this method, multiple iterations were performed to continuously adjust the die surface geometry until the forming accuracy satisfied the required tolerance. The final compensated die surface and the target surface are compared in Figure 15.

**Figure 16:**
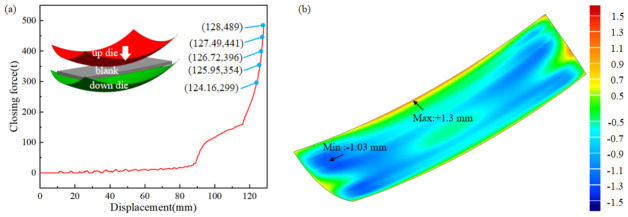
Final clamping force and deviation distribution after compensation: (**a**) clamping force–displacement curve; (**b**) comparison of deviations between the compensated formed part and the target surface.

Figure 16 shows the clamping force and springback distribution at a clamping displacement of 128 mm after compensation. The optimized maximum clamping force is only 489 t (Figure 16a), which is much lower than the maximum clamping force of 5090 t in the unoptimized case (Figure 11a), corresponding to a reduction of approximately 4600 t. After compensation, the deviations are all controlled within the range of −2.0 to 2.0 mm. The maximum springback occurs in the middle region of the two short edges of the blank, while most of the deviations fall within −1.1 to 0.5 mm, accounting for approximately 90.88% of the total. The maximum deviation is only 1.3 mm, and after excluding the machining allowance, the maximum deviation is reduced to 0.96 mm.

**Figure 17:**
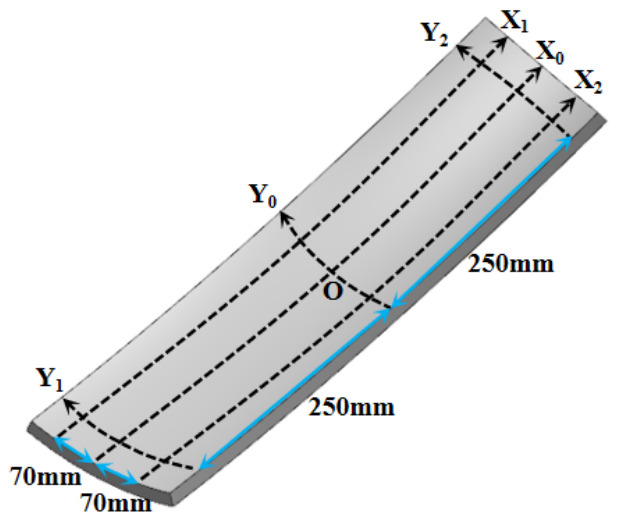
Schematic diagram of typical sectional lines.

To quantitatively evaluate the springback behavior, the deviation of typical sectional profiles of the simulated large-scale high-strength steel doubly curved thick plate was analyzed. The lowest point of the target surface and the formed surface was selected as the reference point and defined as the origin of the coordinate system, ensuring that both geometries were described within a unified coordinate framework. Subsequently, three representative cross-sectional lines were extracted along both the X and Y directions for comparison. The X0 and Y0 sections are mutually perpendicular, and both pass through the lowest point O of the thick plate. The X1 and X2 sections are parallel to X0 with a spacing of 70 mm, where X2 corresponds to the straight edge of the component. The Y1 and Y2 sections are parallel to Y0 with a spacing of 250 mm. The definition of these sectional lines is illustrated in Figure 17.

**Figure 18:**
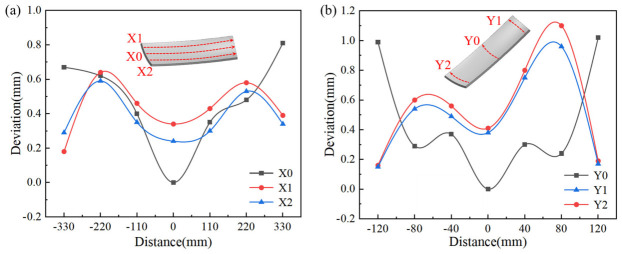
Deviation of typical sectional profiles of the compensated formed part: (**a**) deviation of three representative X-direction sectional lines; (**b**) deviation of three representative Y-direction sectional lines.

The deviation of typical sectional profiles of the compensated large-scale high-strength steel doubly curved thick plate was further analyzed based on the simulation results. The deviations along the X-direction cross-sections are shown in Figure 18, where a significant reduction in overall deviation can be observed. For the X0 section, the deviation exhibits an increasing trend from the middle region toward both ends, reaching a maximum value of 1.08 mm at the edges. The X1 and X2 sections show generally consistent springback behavior, characterized by an initial increase followed by a decrease. The peak deviation occurs at approximately 80 mm away from the origin, with a maximum value of 1.10 mm, and the formed surface in this region is slightly higher than the target surface.

For the Y0 section, the deviation also increases with distance from the origin and reaches its maximum value near the edge region; however, the overall magnitude is lower than that in the X-direction, with a maximum deviation of 0.81 mm. The Y1 and Y2 sections exhibit similar variation trends, with springback showing an initial increase followed by a decrease. The peak deviation occurs at approximately 220 mm from the origin, and the maximum deviation is 0.58 mm.

## 5. Experimental Validation and Discussion

### 5.1. Experimental Equipment and Molds

**Figure 19:**
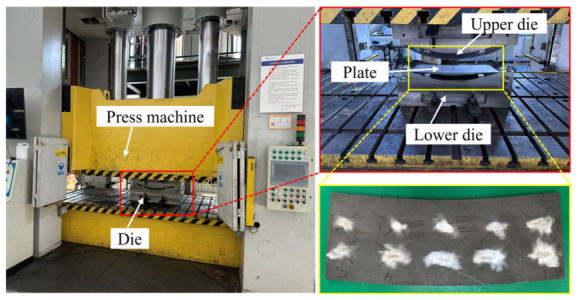
Experimental setup and die.

The integral pressing experiment of the Q890 high-strength steel thick plate was conducted on a DUT 1000 t hydraulic press (Jiangdong Machinery Co., Ltd. Chongqing, China). The equipment has a worktable size of 3500 × 1500 mm, a ram stroke of 1500 mm, and a maximum clamping force capacity of 600 t. The die used in the experiment has dimensions of 900 × 400 × 460 mm and is made of 45# steel, as shown in Figure 19. During the forming process, a force-controlled clamping strategy was adopted to complete the integral pressing operation, in order to meet the high-load forming requirements of the Q890 high-strength steel thick plate component.

### 5.2. Experimental Results and Analysis

**Figure 20:**
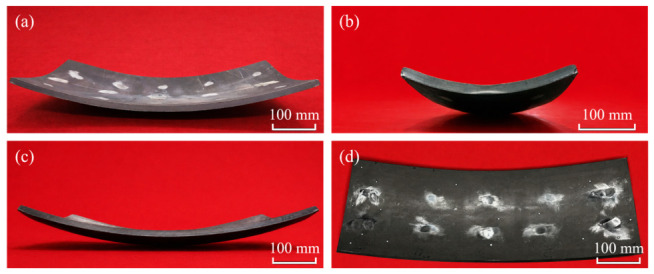
Schematic of the experimental component: (**a**) three-view drawing of the specimen; (**b**) side view; (**c**) front view; (**d**) top view.

After positioning the initial blank, the ram of the hydraulic press was driven downward to move the upper die. Once the upper die came into contact with the blank, loading was continued at a low speed until the system pressure stabilized, entering the dwell-holding stage. The holding time was set to 30 s. Subsequently, the upper die was retracted, and unloading was completed to obtain the formed specimen, as shown in Figure 20.

**Figure 21:**
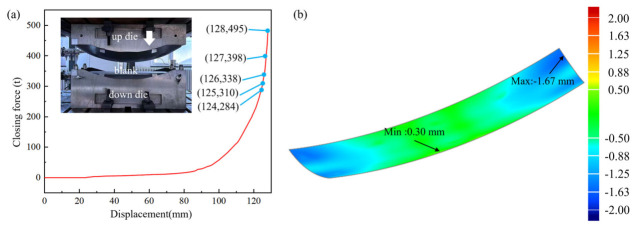
Clamping force and deviation distribution of the experimental component: (**a**) clamping force–displacement curve; (**b**) comparison of deviations between the experimental component and the target surface.

To obtain the springback characteristics of the experimental component, a handheld 3D optical scanner (Hexagon Manufacturing Intelligence, Qingdao, China) was used to measure the inner surface geometry of the formed part. The scanned experimental data and the target inner surface were imported into a 3D reverse engineering verification software (geomagic qualify 2013). The inner surface of the target part was taken as the reference, and only the central region of the component was considered by removing the machining allowance, since only this region was used in the final application. The 3D comparison results are shown in Figure 21. It can be clearly observed that the experimental component exhibits high forming accuracy. The geometric comparison indicates good agreement between the experimental part and the target surface, with a maximum deviation of only 1.67 mm. This satisfies the required forming accuracy specifications.

**Figure 22:**
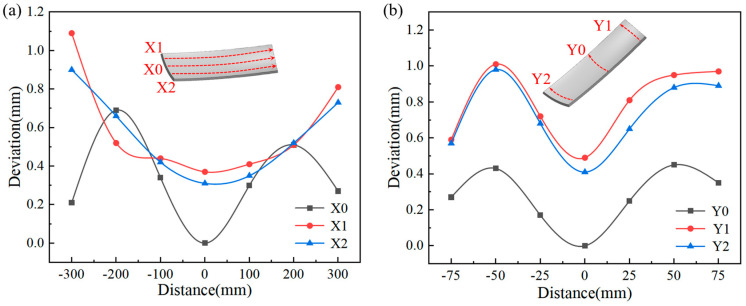
Deviation of typical sectional profiles of the experimental component: (**a**) deviation of three representative X-direction sectional lines; (**b**) deviation of three representative Y-direction sectional lines.

The springback of the experimental component along typical sectional profiles exhibits a distinctly non-uniform distribution, as shown in Figure 22. In the X-direction sections, a characteristic “large at both sides and small in the middle” distribution is observed. For the X0 section, the maximum deviation occurs at both ends, reaching 0.45 mm. The X1 and X2 sections show generally consistent trends, with peak values also occurring near the edge regions, and a maximum deviation of 1.01 mm. This indicates that the central region undergoes a higher degree of plastic deformation, resulting in limited elastic recovery after unloading, whereas the edge regions experience reduced constraints and more complete stress release, leading to more pronounced springback.

The Y0 section also exhibits a similar “large at both sides and small in the middle” distribution, with a maximum deviation of 0.69 mm. The Y1 and Y2 sections follow the same trend, with peak deviations occurring at both ends and a maximum value of 1.09 mm. These results further confirm that near the edges of the blank, the constraint conditions are weakened and the proportion of elastic deformation increases, thereby producing a more significant springback response.

The experimental results show that the springback distribution is generally consistent with that obtained from numerical simulations. The regions exhibiting negative deviation are mainly attributed to discrepancies between the modeling assumptions and the actual processing conditions. First, the finite element analysis is typically based on idealized boundary conditions, such as perfect contact conditions and a stable loading path, whereas in the actual forming process, inevitable fluctuations during loading may occur due to equipment and process instability, leading to localized differences in springback response. Second, in the simulation, the friction coefficient is usually assumed to be constant, while in the real forming process it varies significantly with lubrication conditions, surface roughness, and contact pressure, resulting in strongly nonlinear interface friction behavior. In addition, practical cold forming may involve die assembly errors, gap variations, and limited machine stiffness, all of which can influence the stress distribution and unloading path, thereby affecting springback behavior. Meanwhile, the numerical model still involves certain simplifications in the description of residual stress, material anisotropy, and kinematic hardening behavior, making it difficult to fully capture the true material response under complex loading paths. The combined effect of these factors leads to the observed discrepancies in springback distribution between experiments and simulations.

## 6. Conclusions

This study systematically addresses the two fundamental bottlenecks in integral pressing of thick high-strength steel doubly curved shells: the sharp surge in clamping force and the poor predictability of springback. Taking a 16 mm thick Q890 steel shell as the research object, the through-thickness gradient plasticity and the Bauschinger effect were first characterized, and a mixed hardening model incorporating gradient plasticity was established. To mitigate lateral forces induced by the asymmetric geometry, a blank positioning optimization strategy was proposed. A striking clamping force surge phenomenon was then revealed during the final 1 mm of the stroke, and its root cause—synergistic effects of contact area expansion, frictional resistance buildup, and localized upsetting—was elucidated. Based on this mechanism, a normal-direction over-compensation strategy was developed to intentionally maintain a bending-dominant deformation mode at the target clamping position, thereby avoiding the critical conditions that trigger the force surge. Through iterative die surface optimization, a low-load, high-accuracy forming solution was obtained and successfully validated by experimental pressing on a 1000 t hydraulic press. The main conclusions are as follows:

(1) The Q890 high-strength steel thick plate exhibits pronounced through-thickness gradient plasticity. The yield strength varies from 948.74 MPa at the core to 1008.07 MPa at the surface, corresponding to a maximum difference of approximately 59.33 MPa and a variation ratio of about 6.25%. Meanwhile, the material shows a significant Bauschinger effect during reverse loading. Based on the calibrated mixed hardening parameters, the reverse softening magnitude is approximately 190 MPa, accounting for about 18.8–20.0% of the yield strength. Therefore, for the simulation of large-scale graded doubly curved components, both through-thickness gradient plasticity and the Bauschinger effect must be fully considered, and a mixed hardening model incorporating these effects should be established.
(2) To address the lateral force induced during the integral pressing of asymmetric doubly curved shell structures, a blank positioning optimization strategy was proposed. By optimizing the initial placement of the blank, the maximum lateral force during forming was reduced to within 2 t, satisfying equipment constraints and ensuring process stability as well as die safety.
(3) The phenomenon of a sharp clamping force surge during the fully closed stage was revealed. When the clamping displacement increased from 129 mm to 130 mm, the clamping force rose abruptly from approximately 680 t to 5090 t. This phenomenon was mainly attributed to the combined effects of rapid expansion of the contact area, a significant increase in frictional resistance, and localized upsetting in regions already in contact with the die. Therefore, a strategy based on adjusting the normal-direction compensation amount was proposed to suppress the clamping force surge while maintaining forming accuracy.
(4) The normal-direction compensation method shows strong applicability for the forming of large-scale graded doubly curved Q890 thick plates. After compensation, the maximum surface deviation of the component is reduced from 15.2 mm to 1.3 mm, and the maximum deviation of typical sectional profiles is reduced from 3.43 mm to 0.41 mm, enabling high-precision forming.
(5) Experimental validation demonstrates that, with the proposed method, the maximum clamping force is reduced from 5090 t to 495 t. Meanwhile, the maximum surface deviation of the formed part is reduced from 7.15 mm to 0.96 mm (simulation) and 1.67 mm (experiment). By actively modifying the final-stage deformation mode through die surface compensation, the proposed approach achieves a “low-load–high-accuracy” forming process, providing a new technological paradigm for thick plate forming applications.

## Figures and Tables

**Table 1:** Through-thickness mechanical property distribution of the steel plate. (The through-thickness coordinate z is measured from the mid-thickness plane. Int. 1, int. 2, int. 3, and int. 4 correspond to z = 8, 3, −3, and −8 mm, respectively, and denote yield strength obtained by interpolation or extrapolation. Meas. 1, meas. 2, and meas. 3 correspond to z = 6, 0, and −6 mm, respectively, and denote experimentally measured positions).

	Int. 1	Meas. 1	Int. 2	Meas. 2	Int. 3	Meas. 3	Int. 4
yield strength $R_{eH}$/MPa	974.82	968.30	958.52	948.74	970.99	993.24	1008.07
n	0.031	0.0329	0.0306	0.0295	0.0321	0.0352	0.0364

**Table 2:** Parameters of the Chaboche mixed hardening model for Q890 high-strength steel.

Material	Q	b	$c_{1}$	$\gamma_{1}$	$c_{2}$	$\gamma_{2}$	$c_{3}$	$\gamma_{3}$
Q890	−190	0.85	158,865.8	1741.7	20,750.3	824.5	5778.8	33.8

## Data Availability

The original contributions presented in this study are included in the article. Further inquiries can be directed to the corresponding authors.

## References

[1] Ji H., Gao S., Tang M., Yu B., Li X. (2024). Research on point pressing process of curved parts of thick plates with asymmetric double curvature of high strength steel. J. Phys. Conf. Ser..

[2] Kato T., Hirose S., Maeda S., Ikushima K., Tango Y., Notsu A. (2024). Development of AI Line Heating System and Its Application to Automatic Production of Arbitrary Shaped Steel Plate. Q. J. Jpn. Weld. Soc..

[3] Khandai B.K., Gopinath M. (2025). Modelling and monitoring of scaling effects in multi-scan laser forming. Opt. Laser Technol..

[4] Hwang Y.S., Lee H.J., Yang S.Y. (2010). Springback adjustment for multi-point forming of thick plates in shipbuilding. Comput. Aided Des..

[5] Xu H., Liu Y., Zhong W. (2012). Three-dimensional finite element simulation of medium thick plate metal forming and springback. Finite Elem. Anal. Des..

[6] Chongthairungruang B., Uthaisangsuk V., Suranuntchai S., Jirathearanat S. (2013). Springback prediction in sheet metal forming of high strength steels. Mater. Des..

[7] Souza D.T., Rolfe B. (2013). Understanding robustness of springback in high strength steels. Int. J. Mech. Sci..

[8] Lu Z., Li D., Cao L. (2023). Springback control in complex sheet-metal forming based on advanced high-strength steel. Coatings.

[9] Li X.Q., Dong H.R., Yu C.V., Wang H.B., Yang Y.F., Song B.Y. (2020). Influence of yield criteria and hardening model on draw-bending springback prediction of DP780. J. Mech. Eng..

[10] Ben Said L., Bouhamed A., Wali M., Kamoun T., Alhadri M., Ayadi B. (2025). Anisotropic plasticity in sheet metal forming: Experimental and numerical analysis of springback using U-bending test. Machines.

[11] Hou Y., Min J., Lin J., Liu Z., Carsley J.E., Stoughton T.B. (2017). Springback prediction of sheet metals using improved material models. Procedia Eng..

[12] Choi J., Lee J., Bong H.J., Lee M.G., Barlat F. (2018). Advanced constitutive modeling of advanced high strength steel sheets for springback prediction after double stage U-draw bending. Int. J. Solids Struct..

[13] Zhang C., Stiebert F., Wang S., Traphöner H., Niu C., Zhou L. (2025). Springback prediction of advanced lightweight sheet metal considering evolving plastic behaviors under different reverse loadings. J. Manuf. Process..

[14] Jung J., Jun S., Lee H.S., Kim B.M., Lee M.G., Kim J.H. (2017). Anisotropic hardening behaviour and springback of advanced high-strength steels. Metals.

[15] Trzepiecinski T., Lemu H.G. (2017). Effect of computational parameters on springback prediction by numerical simulation. Metals.

[16] Lee J.W., Lee M.G., Barlat F. (2012). Finite element modeling using homogeneous anisotropic hardening and application to spring-back prediction. Int. J. Plast..

[17] Papeleux L., Ponthot J.P. (2002). Finite element simulation of springback in sheet metal forming. J. Mater. Process. Technol..

[18] (2010). Metallic Materials—Tensile Testing—Part 1: Method of Test at Room Temperature.

[19] Geng Y., Lin Y., Wang S., Xu E., He Z., Chen K. (2023). Experiment for measuring mechanical properties of high-strength steel sheets under cyclic loading by V-shaped double-shear-zone specimens. Materials.

[20] Yuan S., Fan X. (2019). Developments and perspectives on the precision forming processes for ultra-large size integrated components. Int. J. Extrem. Manuf..

[21] Zhao J., Zhou G., Zhang D., Kovacic I., Zhu R., Hu H. (2023). Integrated design of a lightweight metastructure for broadband vibration isolation. Int. J. Mech. Sci..

[22] Zhou W., Dong P., Lillemäe I., Remes H. (2020). Analytical treatment of distortion effects on fatigue behaviors of lightweight shipboard structures. Int. J. Fatigue.

[23] Huang X., Yuan Y., Zhao J., Wei C. (2022). Comparative study on ultra-low-cycle-fatigue behavior of Q235 normal-steel and Q690 high-strength steel. J. Constr. Steel Res..

[24] Zhang L., Hou Y., He R., Ye Y., Niu C., Min J. (2022). Machine learning for extending capability of mechanical characterization to improve springback prediction of a quenching and partitioning steel. J. Mater. Process. Technol..

